# Blowout fracture-associated orbital cellulitis progressing to panophthalmitis: a case report

**DOI:** 10.1186/s12886-021-02153-5

**Published:** 2021-11-10

**Authors:** Atsuhide Takesue, Yosuke Asada, Hiroki Ooya, Toshiyuki Yokoyama

**Affiliations:** Department of Ophthalmology, Juntendo Nerima Hospital, 3-1-10 Takanodai, Nerima-ku, Tokyo, 177-8521 Japan

**Keywords:** Orbital cellulitis, Blowout fracture, Eye enucleation, Panophthalmitis, *Parvimonas micra*

## Abstract

**Background:**

*Parvimonas micra* is known as a causative agent of chronic periodontal disease. This Gram-positive obligate anaerobic coccus was cultured from the ocular surface of blowout fracture-related orbital cellulitis progressing to panophthalmitis.

**Case presentation:**

The patient was a woman in her fifties who had panic disorder and subsequently was a victim of domestic violence. These factors led to delayed consultation. At the initial visit to an ophthalmologist, the ocular surface of the right eye was covered with pus. Swelling of the upper and lower eyelids prevented the eyelid from closing and exophthalmos, severe corneal ulcer, panophthalmitis, and no light perception were observed. Head computed tomography revealed an old blowout fracture and chronic sinusitis with orbital cellulitis. *P. micra* were isolated from culture of pus samples from the sinus and from the ocular surface.

**Conclusions:**

There is a possibility that *P. micra* invaded the orbit via the fragile bony site and caused orbital cellulitis, severe corneal ulcer, and panophthalmitis that required enucleation. In cases of coexisting old blowout fracture and chronic sinusitis, the chronic sinusitis should be treated as quickly as possible.

## Background

The indications for and timing of surgical repair of blowout fracture have long been controversial among ophthalmologists, otolaryngologists, and plastic surgeons. Recently, spontaneous radiologic improvement in addition to spontaneous clinical improvement was documented in patients with blowout fracture who had been treated conservatively [1]. Generally, blowout fracture-related orbital cellulitis can occur following paranasal sinus infection preexisting or occurring within several weeks after the fracture. It has been presumed that fracture of the orbital floor may impede blood supply to the inferior orbital fat, leading to anaerobic cellulitis [2]. Meanwhile, since the advent of effective antibiotics, the incidence of severe complications of orbital cellulitis including vision loss, cavernous sinus thrombosis, meningitis, frontal lobe abscess, osteomyelitis, and encephalitis has decreased.

We encountered a case of blowout fracture-related orbital cellulitis with severe corneal ulcer and panophthalmitis that eventually needed enucleation. *Parvimonas micra* (previously known as *Micromonas micros* or *Peptostreptococcus micros*) is the common causative agent of periodontitis and was cultured from pus samples from the sinus and from the ocular surface.

We present this case to raise awareness that eventual panophthalmitis can occur after blowout fracture in the presence of preexisting paranasal sinus infection and delayed specialist consultation.

## Case presentation

**Fig. 1:**
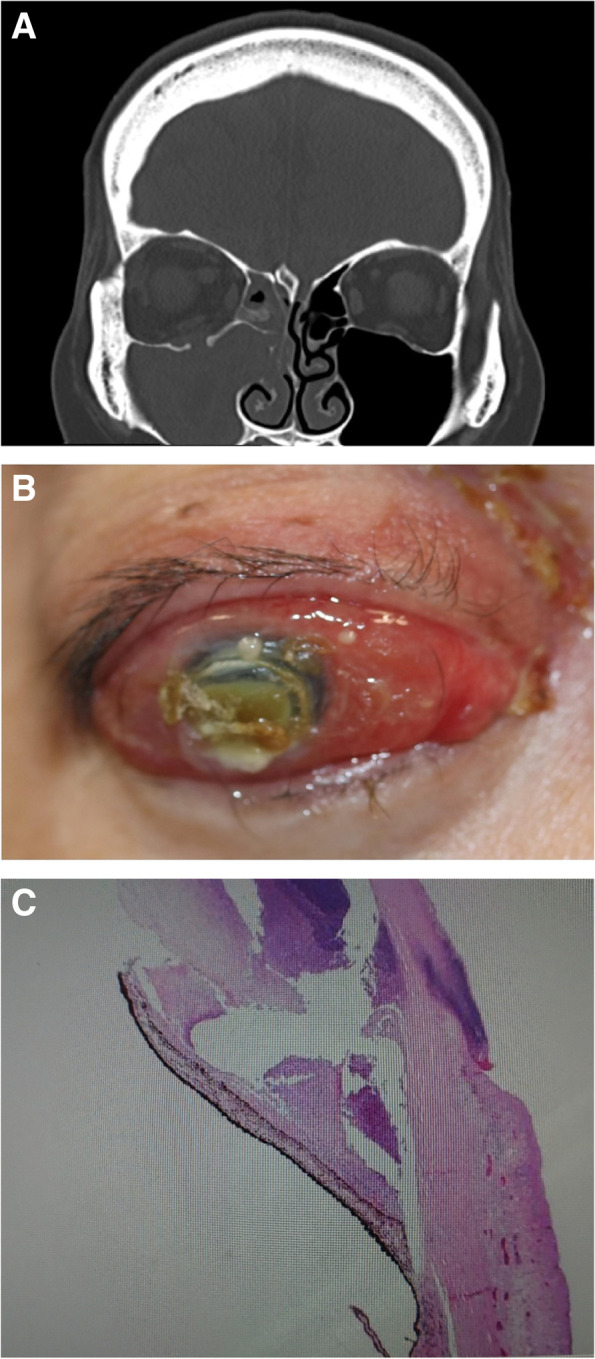
**A** Computed tomography scan of the head showing an old right-sided blowout fracture and sinusitis extending into the inferior of the orbit and into the maxillary and ethmoidal sinuses. **B** Clinical photograph showing exophthalmos, severe corneal ulcer, and panophthalmitis. **C** Hematoxylin and eosin stain of the enucleated eyeball showing large abscess in the anterior chamber

The patient was a woman in her fifties who sustained a head injury as a result of DV by her husband. She was transported by ambulance to the emergency department of our hospital. Her medical history included panic disorder and sleep disorder. Computed tomography (CT) revealed a right orbital floor fracture and sinusitis extending into the inferior of the orbit and into the maxillary and ethmoidal sinuses (Fig. 1A). The orbital floor fracture was judged by a radiologist to be an old fracture. An emergency physician explained to the husband the need for her to get an eye checkup for ocular symptoms because of the old blowout fracture.

Forty-three days after the first visit to our hospital, she was hit again by the husband but continued to endure eye pain at home by using over-the-counter drugs, specifically analgesics, eye drops, and eye ointments. The sharp right eye pain and periorbital swelling gradually deteriorated, and pus began to appear from the right eye. Twenty-eight days following second violence, she accepted to undergo ophthalmological examination at our hospital. On presentation to us, the ocular surface of the right eye was covered with pus. Swelling of the upper and lower eyelids made eyelid closure impossible, and exophthalmos, severe corneal ulcer, panophthalmitis, and no light perception were observed (Fig. 1B). Although antianxiety and antipsychotic drugs were used under the direction of a psychiatrist as she started having a panic attack in the consultation room, ultrasonography and electroretinography could not be carried out. There was no obvious bruising around the eye and no tear on the ocular surface at that time. Head CT showed the old blowout fracture and chronic sinusitis as observed previously, but also orbital cellulitis and maxilla fracture that had not been previously detected. Orbital CT also showed that the shape of eyeball was maintained and that there was neither foreign body nor abscess in the orbit. It was noteworthy that there was no new blowout fracture. These findings raised concerns that orbital cellulitis could progress to intracranial infection unless intensive treatment was started. These potential risks were explained to her and the husband, but consent to immediate admission could not be obtained. She also strongly insisted on returning to the marital home and did so. Gram stain of pus sample obtained from the ocular surface showed gram-positive cocci, gram-positive bacilli, and gram-negative bacilli. In addition, *Mitis. streptococcus* and *P. micra* were isolated on anaerobic culture, with *gamma-streptococci* on aerobic culture.

The patient returned to our hospital at thirty-five days following second violence due to intolerable pain. As it seemed the topical and oral levofloxacin hydrate (Cravit®) had been ineffective, we recommended enucleation for the purposes of reducing the pain as quickly as possible and preventing orbital cellulitis from progressing to encephalitis. After obtaining her consent, surgical drainage and irrigation of the sinuses and enucleation were performed on the same day. Examination of the enucleated eyeball confirmed large abscess in the anterior chamber and infiltration of neutrophils into the interstitium of the cornea and conjunctiva (Fig. 1C). From this pathological result, we considered that orbital cellulitis had progressed to panophthalmitis. Gram stain of a pus sample from the maxillary paranasal sinus at surgical drainage yielded gram-positive cocci, gram-positive bacilli, gram-negative bacilli, and leucocytes. Again, *M. streptococcus* and *P. micra* were isolated on anaerobic culture.

Postoperatively, following intervention by the police and medical social workers, the psychiatrist proposed that the patient be transferred to the psychiatric hospital secretly and be kept under isolation from the husband. Nevertheless, she showed strong intention to return home with the husband and did so. After the pain resolved, she was referred to the department of plastic surgery at another university hospital to receive a prosthetic eye.

## Discussion and conclusion

A large study investigating the frequency of orbital cellulitis-associated encephalitis found that 9 (4.1%) of 218 patients diagnosed with severe orbital cellulitis had accompanying intracranial extension [3]. In our case, in addition to preventing encephalitis, alleviating pain was a priority, and therefore enucleation was performed.

Regarding the frequency of blowout fracture-related orbital cellulitis, there are two valuable reports. One found that out of 83 patients who had been hospitalized due to orbital complications of sinusitis, 5 (6.2%) had experienced blowout fracture in the past 3 weeks [4]. Of these 5 cases, however, it was unclear whether the chronic sinusitis had developed before the fracture. The other report found that 4 (0.8%) of 497 patients with blowout fracture had accompanying orbital cellulitis, and of these 4, only 2 had a history of upper respiratory tract infection before the orbital fracture [5]. Generally, vison loss as a consequence of inflammatory paranasal sinus disease is a rare but well-recognized complication. Several mechanisms have been proposed to explain paranasal sinus disease-associated vision loss: (1) optic neuritis as a result of an adjacent inflammatory process; (2) venous congestion of the optic nerve due to raised intraorbital pressure or thrombophlebitis of the veins; and (3) raised intraorbital pressure resulting in central retinal artery occlusion [6].

However, the clinical progress of our patient seems to be different from the mechanism described above. After the orbital cellulitis, the orbital infection was left untreated for 4 weeks because of the prevailing circumstances. One was the repeated episodes of DV. Victims of DV are often hesitant to seek medical help and are ashamed to disclose their situation or afraid of being ridiculed or ignored [7]. The other was panic disorder. She feared visiting the hospital and accepting medical intervention due to pathological anxiety. The failure of her receiving urgent treatment is attributed to these two factors. Thus, there is a possibility that blowout fracture-associated orbital cellulitis developed to blepharitis, exophthalmos, conjunctivitis, severe corneal ulcer, and panophthalmitis because there had been no obvious bruising around the eye, no tear on the ocular surface, and no medical history of dacryocystitis. Considering that blowout fracture is not basically accompanied by corneal ulcer or panophthalmitis, the possibility that traumatic corneal ulcer had been first developed and led to panophthalmitis was low. In fact, it is reported that out of 268 patients who underwent surgical repair for blowout fracture, there were no cases of corneal ulcer nor panophthalmitis [8].

*P. micra* were detected at both the ocular surface and the sinus. *P. micra* is a Gram-positive obligate anaerobic coccus, and is known as a causative agent of chronic periodontal disease, peritonsillar abscess, chronic suppurative otitis media, lung abscess, and chronic sinusitis. To our knowledge, there is only one report of *P. micra* cultured from ocular surface: refractory purulent conjunctivitis associated with *P. micra* [9]. However, this is an exceptional case and the authors did not explain how the conjunctiva got infected with *P. micra*. As a matter of fact, a previous Japanese study that investigated the isolation rate of pathogenic bacteria in 1378 cases of external ocular bacterial infection did not report the presence of *P. micra* [10].

Therefore, *P. micra* was presumed to have caused chronic sinusitis and invaded the orbit via the fragile bony site from the blowout fracture, which had been made more vulnerable by repeated physical trauma. Then, the bacteria might have caused orbital cellulitis and eventually led to panophthalmitis. To our knowledge, there is only one report on blowout fracture-associated orbital cellulitis progressing to panophthalmitis, although the authors were unable to culture identical species of bacteria from both the sinus and ocular site [11].

In cases of coexisting old blowout fracture and chronic sinusitis, and where repeated blunt orbital trauma is unfortunately predicted, it is preferable to treat chronic sinusitis as soon as possible.

## Data Availability

All data generated or analyzed during this study are included in this published article.

## Abbreviations

CT: Computed Tomography
DV: Domestic Violence
*P. micra*: *Parvimonas micra*;
*M. streptococcus*: *Mitis streptococcus*
